# Artificial Intelligence Adoption as a Driver of Innovation and Competitiveness in SMEs: A Bibliometric and Systematic Review

**DOI:** 10.12688/f1000research.171494.1

**Published:** 2025-10-31

**Authors:** Yohannes Yesuf, Ziska Fields

**Affiliations:** 1 Business Management, University of Johannesburg College of Business and Economics, Auckland Park, Gauteng, South Africa

**Keywords:** Adoption, artificial intelligence, entrepreneurial decision-making, innovation, small and medium-sized enterprises

## Abstract

This study employs a bibliometric and systematic literature review (B-SLR) approach, guided by the PRISMA 2021 framework, to investigate the transformative impact of Artificial Intelligence (AI) on small and medium-sized enterprises (SMEs). Drawing on peer-reviewed studies published between 2020 and 2024 across the Scopus databases, the review integrates quantitative mapping and qualitative synthesis to identify emerging research patterns, thematic concentrations, and methodological trends. The findings reveal a marked surge in scholarly engagement since 2020, reflecting growing recognition of AI as both a strategic and operational driver of competitiveness in SMEs. Three dominant thematic clusters emerge: (1) AI-driven innovation, encompassing applications such as machine learning, predictive analytics, automation, and process optimization; (2) entrepreneurial decision-making, highlighting how AI supports opportunity recognition, strategic planning, and market sensing; and (3) adoption barriers and enablers, focusing on organizational readiness, digital infrastructure, human capital, and financial capability. While the field demonstrates expanding theoretical and practical interest, significant gaps remain regarding methodological diversity, contextual variation across developing economies, and the integration of socio-ethical considerations in AI adoption. The study contributes by consolidating fragmented knowledge, mapping the intellectual structure of the domain, and offering actionable implications for policymakers, practitioners, and scholars seeking to leverage AI to foster innovation, digital resilience, and sustainable growth in SMEs.

## Introduction

Small and Medium-sized Enterprises (SMEs) constitute the backbone of global economies, serving as key contributors to innovation, employment generation, and sustainable growth across advanced and emerging markets ( Ndubisi et al., 2021). Accounting for nearly 90% of global businesses and more than half of total employment, SMEs are indispensable in fostering entrepreneurship and driving socio-economic dynamism ( World Bank Group, 2022). Beyond their economic significance, SMEs are vital engines of innovation, often experimenting with novel products, services, and business models to respond to rapidly shifting customer demands and competitive pressures ( Albats et al., 2023). However, their innovation capacity is frequently constrained by structural and operational challenges, such as limited financial resources, weak technological capabilities, managerial gaps, and inadequate access to advanced digital infrastructures ( Wang et al., 2025). These barriers hinder their ability to achieve competitiveness in increasingly digital and knowledge-driven economies.

The advent of Artificial Intelligence (AI) presents a transformative pathway for SMEs to address these limitations and enhance their competitiveness ( Iyelolu et al., 2024). AI technologies, including machine learning, natural language processing, computer vision, and predictive analytics, equip firms with tools to automate processes, improve decision-making, and generate innovative solutions that strengthen both operational efficiency and market responsiveness ( Ghobakhloo et al., 2023). In the SME context, AI adoption has been shown to accelerate product development, deepen customer engagement, strengthen market intelligence, and improve strategic decision-making ( Iyelolu et al., 2024). Moreover, AI enables the recognition of opportunities and evidence-based entrepreneurial choices, making it a powerful driver of innovation and competitiveness, especially in uncertain and resource-constrained environments ( Giuggioli & Pellegrini, 2023).

Nevertheless, AI adoption within SMEs is neither uniform nor straightforward. While some enterprises have successfully embedded AI into their operations, many remain at the experimental or exploratory stage, largely due to organizational, technological, and institutional barriers ( Khan et al., 2025). Factors such as digital readiness, access to financial and human capital, and strategic alignment are critical in determining the success of adoption initiatives ( Omowole et al., 2024). Similarly, external pressures, including regulatory environments, market competition, and access to digital ecosystems, strongly shape adoption trajectories ( Zahra et al., 2023). This heterogeneity underscores the need for a systematic examination of both the drivers and barriers to AI adoption across diverse contexts.

Although scholarly attention to AI adoption in SMEs has expanded, research remains fragmented and unevenly distributed across disciplinary and geographical boundaries ( Ayinaddis, 2025; Siddiqui et al., 2024). Much of the existing work focuses on technical implementations or single-case explorations, providing limited insights into broader theoretical, methodological, and practical patterns ( Schwaeke et al., 2025). Furthermore, most studies disproportionately emphasize developed economies, leaving SMEs in emerging economies, where challenges and opportunities are distinct, relatively underexplored ( OECD, 2024). This imbalance restricts the generalizability of findings and limits the development of context-sensitive strategies for the adoption of AI.

To bridge these gaps, this study integrates bibliometric and systematic literature review (B-SLR) approaches to provide a comprehensive overview of the intellectual landscape on AI adoption in SMEs. Bibliometric analysis enables the identification of key research trends, influential authors, collaboration networks, and thematic clusters ( Hassan & Duarte, 2024), while the SLR approach synthesizes empirical evidence, highlights methodological tendencies, and identifies critical barriers, enablers, and innovation outcomes ( Marzi et al., 2025). Together, these methods provide a dual lens for mapping the evolution of the field and advancing theoretical and practical insights. This study specifically addresses the question: How has the adoption of AI in SMEs evolved over time? What dominant themes and methodologies characterize the field? What factors drive or hinder AI adoption, and how do these shape innovation and competitiveness outcomes?

In doing so, this research makes three contributions. First, it enriches academic understanding by systematically mapping the technological, organizational, and environmental determinants of AI adoption in SMEs and linking them to outcomes of innovation and competitiveness. Second, it provides practitioners with actionable insights on how AI can be leveraged to enhance process efficiency, product innovation, and market positioning. Third, it provides policymakers with evidence-based guidance on designing supportive infrastructures, funding mechanisms, and regulatory frameworks that facilitate inclusive AI-driven growth, particularly in resource-constrained and emerging contexts. Ultimately, the study advances the discourse on AI adoption in SMEs by integrating fragmented scholarship, uncovering research gaps, and setting an agenda for future studies at the intersection of AI, entrepreneurship, and innovation.

### AI Adoption, innovation, and competitiveness

AI adoption is increasingly recognized as a catalyst for innovation and competitiveness in SMEs. However, recent studies caution that the benefits are not automatic and largely depend on organization-specific and contextual conditions. Large-scale surveys and empirical evidence indicate a rapid increase in the use of generative and applied AI across businesses; however, the extent of adoption and its outcomes vary considerably depending on organizational capabilities, sectoral dynamics, and institutional environments ( Arroyabe et al., 2024). The literature highlights three key domains where AI adoption can enhance SME competitiveness: operational efficiency, innovation in products and services, and responsiveness to market demands ( Carayannis et al., 2025; Iyelolu et al., 2024). Process automation and data-driven analytics help reduce costs, streamline production cycles, and enhance decision-making ( Adekunle et al., 2021). Sector-based evidence also indicates notable improvements in productivity and export performance among firms with higher digital maturity and more advanced AI integration ( Segarra-Blasco et al., 2025). Despite this, many SMEs still achieve only modest gains because AI adoption is often limited to narrow applications, such as customer support, scheduling, or marketing, rather than being embedded within broader business model transformation and innovation strategies ( Castillo-Vergara et al., 2025).

To explain these differences, scholars often employ the Technology-Organization-Environment (TOE) framework in conjunction with the dynamic capabilities perspective. From the TOE perspective, technological readiness (e.g., data availability and platforms), organizational preparedness (e.g., leadership commitment and employee skills), and external conditions (e.g., regulatory support and competitive pressure) jointly shape both adoption and its outcomes ( Sánchez et al., 2025). The dynamic capabilities approach further emphasizes absorptive capacity, learning orientation, and agility as mediators in the process of translating AI investment into innovation and competitive advantage ( Ferreira et al., 2021).

Several studies argue that complementary investments are essential for AI adoption to deliver transformative results. Cross-country analyses reveal that SMEs achieve strategic benefits from AI only when investments are made in workforce reskilling, data governance, managerial experimentation, and integration into core business processes ( Arroyabe et al., 2024). Without these, AI tends to generate localized productivity improvements that fail to translate into long-term competitiveness. At the same time, persistent barriers continue to limit meaningful AI adoption among SMEs. Financial and human resource constraints, inadequate digital infrastructure, poor data quality, and insufficient managerial knowledge are frequently reported challenges (e.g., Omowole et al., 2024; Omrani et al., 2022). Industry reports also note that many SMEs do not invest adequately in in-house training, resulting in adoption being concentrated in firms with stronger financial capacity or international linkages ( Nazir et al., 2025).

Ecosystem-level interventions are highlighted as critical in bridging these gaps. European policy analyses indicate that public initiatives, sector-specific incubators, affordable access to cloud platforms, and targeted skills development programs enhance adoption rates and facilitate a shift from basic AI use to innovation-driven integration ( Schneegans et al., 2021). Such initiatives are particularly vital in low-resource settings, where market limitations and coordination failures constrain organizations’ capacity to invest in digital capabilities. Nonetheless, emerging critical perspectives caution against overly optimistic assumptions. Recent industry surveys indicate that many generative AI pilots underperform, primarily due to a lack of alignment with organizational strategy and inadequate governance structures ( Feher, 2025). This reinforces the argument that strong leadership, effective change management, and robust governance mechanisms are essential in transforming experimental AI adoption into a sustainable competitive advantage ( Kulkov et al., 2024).

Overall, the latest scholarship underscores that while AI can serve as a powerful enabler of innovation and competitiveness for SMEs, its success depends on complementary capabilities within the organizations, supportive institutional ecosystems, and deliberate strategic leadership. Policy measures that enhance digital skills, strengthen data infrastructure, and provide tailored implementation support appear particularly effective in enabling SMEs to translate AI adoption into durable innovation outcomes.

## Methodology

This study adopts a dual methodological approach, B-SLR, to investigate AI adoption in SMEs. The rationale for this combined approach lies in the complementary strengths of the two methods: bibliometric analysis provides a quantitative, macro-level understanding of research trends, influential authors, collaboration networks, and thematic structures, whereas SLR offers a qualitative, in-depth synthesis of empirical findings, methodological approaches, and theoretical contributions ( Aria & Cuccurullo, 2017; Marzi et al., 2025). Integrating these methods enhances the validity, comprehensiveness, and interpretive richness of the study, allowing it to capture both the breadth and depth of scholarship on AI adoption in SMEs. Such a dual approach is particularly justified in fragmented research domains, where studies are dispersed across disciplines like management and entrepreneurship, and where insights from one method alone may provide an incomplete picture ( Marzi et al., 2025).

### Bibliometric analysis

Bibliometric analysis is widely recognized for its rigor in systematically mapping scientific knowledge and identifying intellectual structures within a research field ( Donthu et al., 2021). In this study, publications were retrieved from Scopus, selected for their extensive coverage, high indexing quality, and broad representation of peer-reviewed scholarly outputs. The keyword strategy, incorporating terms such as “Artificial Intelligence,” “AI adoption,” “SMEs,” “small and medium enterprises,” and “entrepreneurship,” was designed to capture literature reflecting both technological applications and managerial implications. The temporal scope of 2020-2024 was chosen to encompass the evolution of AI research from early conceptual explorations to recent developments influenced by Industry 4.0, digital transformation, and emerging AI-driven business models.

After cleaning the dataset to remove duplicates and irrelevant records, a bibliometric analysis was conducted using VOSviewer. The software constructs and visualizes networks of co-authorship, co-citation, and keyword co-occurrence, offering clear representations of intellectual structures, thematic clusters, and research frontiers. This approach provides systematic and replicable insights into influential scholars, institutions, emerging themes, and underexplored areas in the adoption of AI by SMEs. The use of bibliometric techniques is therefore justified, as they enable evidence-based identification of research frontiers, knowledge gaps, and intellectual structures, ensuring a comprehensive overview of this rapidly expanding field ( Kraus et al., 2024).

### Systematic Literature Review (SLR)

The SLR complements the bibliometric mapping by providing qualitative depth, synthesizing empirical findings, methodologies, and theoretical perspectives related to AI adoption in SMEs. The SLR followed PRISMA guidelines ( Page et al., 2021), ensuring transparency, reproducibility, and methodological rigor. This approach is particularly justified given the fragmented nature of AI adoption research, which spans multiple disciplines, sectors, and geographies. By systematically evaluating existing studies, the SLR helps identify factors influencing adoption, innovation outcomes, and barriers/enablers, while providing a theoretical and practical lens on managerial decision-making and competitiveness.

**Figure 1.**
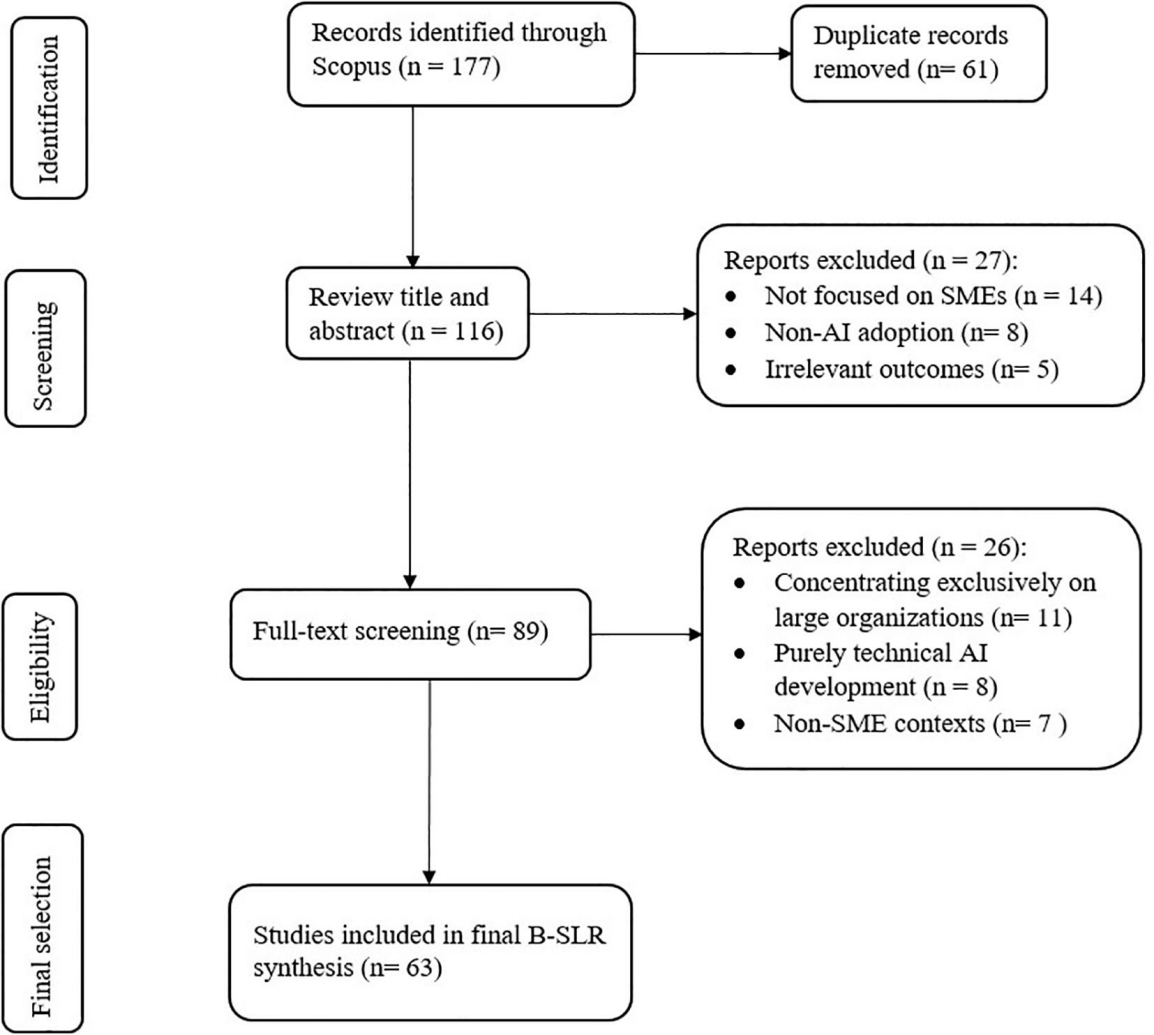
PRISMA framework for this B-SLR, adapted from Page et al. (2021).

The review process followed a structured multi-stage approach to ensure rigor and reliability. In the identification stage, a total of 177 peer-reviewed journal articles published in English were retrieved from Scopus using the same keyword strategy applied in the bibliometric analysis. Grey literature, conference proceedings, and non-peer-reviewed sources were excluded to maintain the research’s quality and integrity. During the screening stage, titles and abstracts were reviewed, resulting in the retention of 89 studies that empirically examined AI adoption in SMEs with a focus on organizational, managerial, or entrepreneurial aspects. In the eligibility assessment, full texts were assessed against predefined inclusion criteria, and studies concentrating exclusively on large organizations, purely technical AI development, or non-SME contexts were excluded. This refinement justified the focus on SMEs as distinct organizational entities with unique challenges, leaving 63 eligible articles for analysis (see Table 1 and Figure 1). Finally, in the data extraction stage, structured information was captured from each study, including publication year, regional and sectoral context, research objectives and questions, methodological approaches (qualitative, quantitative, or mixed methods), AI focus (technology type and application domain), and key findings related to innovation, entrepreneurial decision-making, and competitiveness outcomes.

**Table 1.** Overview of the B-SLR identification and screening process.

Phase	Description	Records (n)
**Identification**	Records identified through Scopus using a keyword strategy	177
	Records after duplicates removed	116
**Screening**	Records screened by title and abstract	89
	Records excluded (e.g., not related to SMEs, irrelevant scope)	67
**Eligibility**	Full-text articles assessed for eligibility	89
	Full-text articles excluded (e.g., large organizations only, purely technical focus, not empirical)	26
**Included**	Studies included in the final synthesis	63

Thematic synthesis was employed to code the extracted data and identify key themes, including AI-driven innovation, adoption enablers and barriers, and managerial strategies. Combining quantitative bibliometric mapping with qualitative thematic analysis allowed for a comprehensive understanding of SMEs’ adoption of AI. This dual approach highlighted the field’s intellectual structure and context-specific insights, including sectoral differences, geographical gaps, and adoption drivers. The methodology strengthens reliability and practical relevance, providing a solid basis to guide future research, support managerial decisions, and inform policies that promote AI-driven innovation and competitiveness in SMEs.

## Results

The bibliometric analysis reveals a clear and accelerating trajectory of scholarly interest in AI adoption in SMEs over the past two decades. Between 2010 and 2015, research on this topic was sparse and largely conceptual, focusing on theoretical models of technology adoption and organizational readiness ( Cunningham et al., 2023). Studies during this period often extrapolated findings from larger firms to SMEs, with limited empirical validation. From 2016 to 2020, the number of publications increased moderately, reflecting the early adoption of Industry 4.0 initiatives and the growing recognition of digital transformation as a strategic priority for SMEs ( Kee et al., 2025; Pelletier & Cloutier, 2019; Yaqub & Alsabban, 2023). Post-2020, there has been a marked surge in publications, coinciding with widespread AI integration into business operations and heightened attention to innovation, strategic planning, and competitive advantage ( Carayannis et al., 2025). This trend highlights both the growing academic interest and the alignment of research with practical business needs in digitally evolving economies.

**Figure 2.**
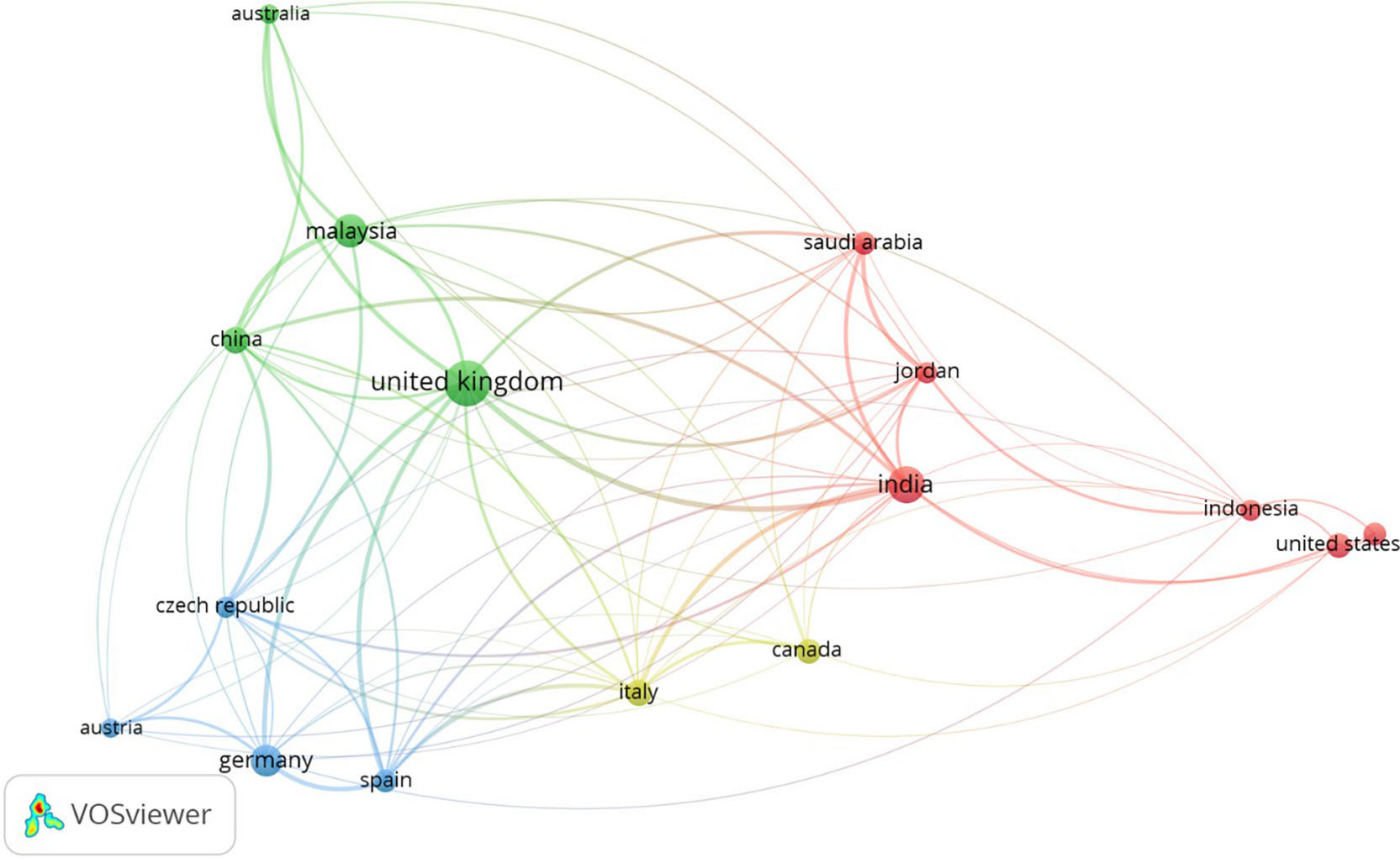
Bibliographic coupling analysis of country-level contributions.

**Figure 3.**
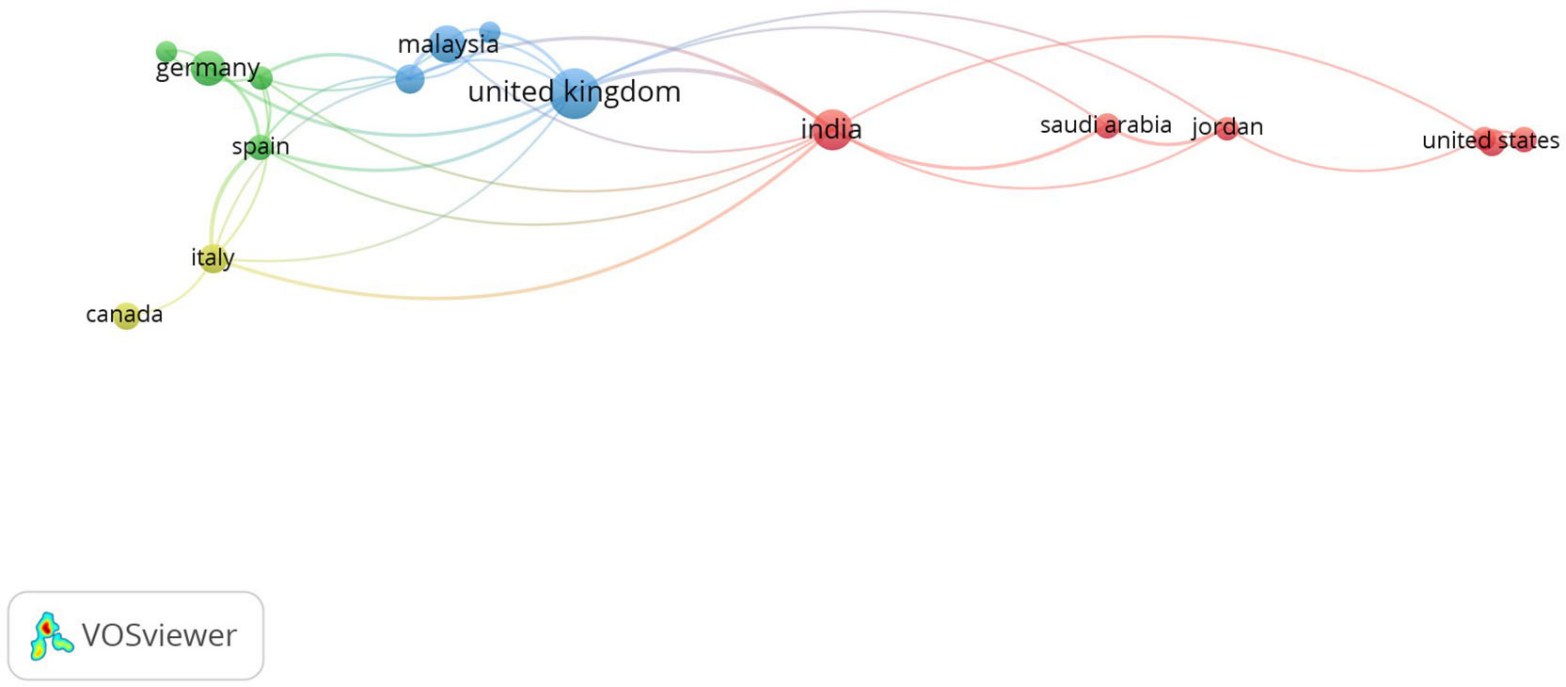
Co-authorship networks across countries.

Bibliographic coupling analysis at the country level reveals that scholarship in the field is largely concentrated in Europe and North America, with notable contributions from the United Kingdom, Germany, the United States, and Canada ( Ayinaddis, 2025; Oldemeyer et al., 2025). Patterns of co-authorship suggest the expansion of collaborative research networks; however, the involvement of emerging economies remains limited (see Figures 2 and 3). This regional imbalance underscores a significant knowledge gap regarding SMEs operating within contexts characterized by resource constraints, regulatory diversity, and limited digital infrastructure ( Aderibigbe et al., 2023).

**Figure 4.**
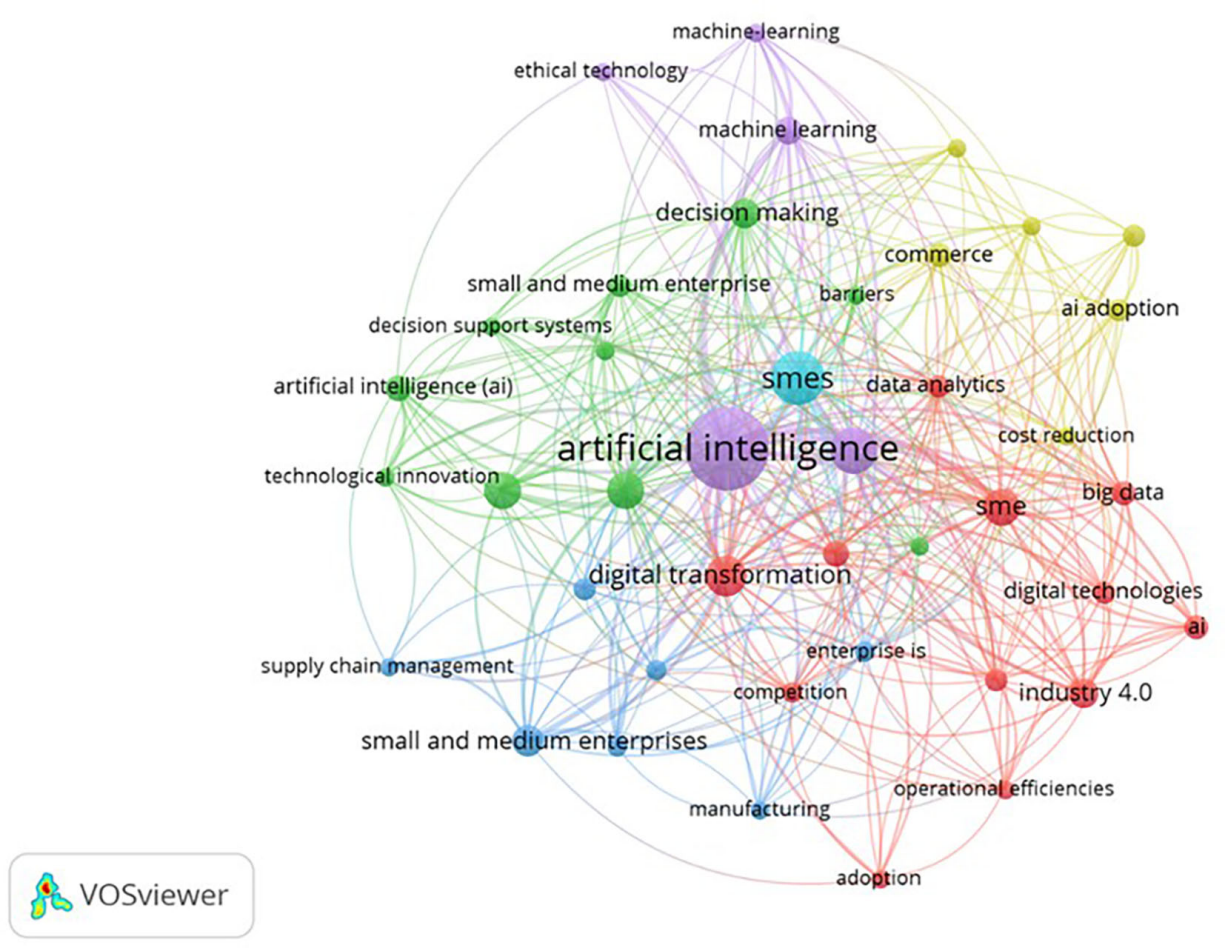
Keyword co-occurrence mapping.

Keyword co-occurrence and thematic mapping identify three principal clusters (see Figure 4 and Table 2). The first, AI-driven innovation, highlights the contribution of machine learning, predictive analytics, and process automation to improving operational efficiency, product development, and competitiveness ( Jalil et al., 2025; Wei & Pardo, 2022). The second, entrepreneurial decision-making, emphasizes how AI facilitates opportunity recognition, evidence-based strategic planning, and agile responses to market uncertainty ( Ikbal, 2025; Vudugula et al., 2023). The third, adoption barriers and enablers, encompasses internal and external factors, such as human capital, technological infrastructure, regulatory conditions, and integration into digital ecosystems ( Prasad Agrawal, 2024; Trevisan et al., 2023). Studies consistently identify financial constraints, limited digital literacy, and weak organizational support as obstacles to AI adoption. At the same time, leadership commitment, inter-organizational partnerships, and targeted training emerge as key enabling mechanisms.

**Table 2.** Key research clusters and representative keywords.

Cluster	Representative keywords	Focus area
**AI-driven Innovation**	Machine learning, predictive analytics, automation, process optimization	Operational efficiency, product innovation, and competitiveness
**Entrepreneurial Decision-Making **	Opportunity recognition, strategic planning, market sensing	Evidence-based managerial decisions, risk management
**Adoption Barriers/Enablers**	Organizational readiness, human capital, financial resources, and digital ecosystems	Determinants of adoption, enabling strategies

The systematic literature review extends the bibliometric insights by offering empirical evidence on the outcomes of AI adoption. Findings indicate that AI enhances operational efficiency, reduces costs, streamlines supply chains, and improves customer engagement ( Iyelolu et al., 2024). Furthermore, AI catalyzes process and product innovation, supporting SMEs in developing new offerings, reconfiguring business models, and achieving competitive differentiation ( Sjödin et al., 2023; Tetteh et al., 2025). It also enhances entrepreneurial decision-making by leveraging predictive analytics and real-time insights for market sensing, risk assessment, and informed strategic investment. Nonetheless, the impact of AI adoption is highly contingent upon factors such as firm size, industry characteristics, managerial capabilities, and regional context ( Horani et al., 2025).

Methodologically, the empirical literature is dominated by case studies and survey-based research, which account for the majority of existing studies. In contrast, longitudinal and experimental approaches remain limited, constraining insights into the long-term effects of AI adoption on innovation and competitiveness ( Schwaeke et al., 2025). Moreover, the evidence base disproportionately centers on SMEs in Europe and North America, with considerably less attention given to those in Asia, Africa, and Latin America. This geographic imbalance underscores the need for context-sensitive research that addresses the technological, institutional, and resource-related challenges faced by SMEs in emerging economies ( Dey et al., 2024; Kumar et al., 2024).

In conclusion, the combined insights from bibliometric and systematic review analyses offer a comprehensive perspective on AI adoption within SMEs. The findings indicate a growing scholarly focus, with innovation, decision-making processes, and adoption drivers identified as key thematic areas. At the same time, notable methodological and regional limitations remain. These outcomes offer valuable guidance for researchers, practitioners, and policymakers seeking to promote AI-enabled innovation, enhance entrepreneurial decision-making, and boost the global competitiveness of SMEs.

Table 3 provides a structured overview of three central cluster themes in the study of AI adoption in SMEs: AI-driven innovation, entrepreneurial decision-making, and adoption barriers/enablers. Each theme is further broken down into sub-themes that capture specific dimensions of AI application and organizational dynamics. For AI-driven innovation, sub-themes such as machine learning, predictive analytics, automation, and process optimization illustrate the technological mechanisms through which SMEs enhance product, service, and operational innovation. Within the entrepreneurial decision-making cluster, opportunity recognition, strategic planning, and market sensing highlight how AI supports informed, agile, and market-oriented managerial decisions. Finally, the adoption barriers/enablers theme emphasizes organizational readiness, human capital, financial resources, and digital ecosystems as key factors that shape the successful implementation of AI solutions. The table synthesizes key focus areas and representative studies, offering scholars and practitioners a concise framework to understand how AI technologies, entrepreneurial processes, and organizational capabilities interact to drive SME performance.

**Table 3.** Thematic clusters, associated sub-domains, core focus areas, and illustrative studies.

Cluster theme	Sub-themes	Key focus	Representative studies
**AI-driven Innovation**	- Machine Learning - Predictive Analytics - Automation - Process Optimization	Explores how AI technologies enhance innovation through improved processes, automation, and predictive insights.	( Ali Abbasi et al., 2022; Baabdullah et al., 2021; Barata et al., 2023; Belahouaoui & Attak, 2024; Bettoni et al., 2021; Chatterjee et al., 2022; Crockett et al., 2021; Dey et al., 2024; Hui et al., 2024; Le et al., 2020; Mathani et al., 2024; Metin et al., 2024; Musa & Lefkir, 2024; Panigrahi et al., 2023; Parra-Sánchez & Talero-Sarmiento, 2024; Rawashdeh, 2022; Taherizadeh & Beaudry, 2023; Tominc et al., 2024; Wei & Pardo, 2022)
**Entrepreneurial Decision-Making **	- Opportunity Recognition - Strategic Planning - Market Sensing	Examines how AI supports entrepreneurs in identifying opportunities, making strategic choices, and sensing market trends.	( Alaskari et al., 2021; Alsafi & Fan, 2020; Baabdullah et al., 2021; Baldegger et al., 2020; Ejjami, 2024; Enshassi et al., 2024; Etemad, 2023; Hermansyah, 2023; Islam et al., 2024a, b; Kshetri et al., 2025; Magableh et al., 2024; Muminova et al., 2024; Polas et al., 2022; Pozzo et al., 2024; Proenca, 2024; Roux et al., 2023; Saleem et al., 2024; Tawil et al., 2024; Vranda et al., 2021; Zavodna et al., 2024)
**Adoption Barriers/Enablers**	- Organizational Readiness - Human Capital - Financial Resources - Digital Ecosystems	Investigate internal and external factors that facilitate or hinder the adoption of AI in SMEs, including resources, skills, and ecosystem support.	( Aljarboa, 2024; Almashawreh et al., 2024; Anh & Duc, 2024; Arroyabe et al., 2024; Badghish & Soomro, 2024; Chatterjee et al., 2022; Díaz-Arancibia et al., 2024; Fonseka et al., 2022; Govori & Sejdija, 2023; Gupta, 2024; Gupta & Yang, 2024; Hamdan et al., 2022; Jin & Liu, 2025; Kukanja, 2024; Lada et al., 2023; Madhavan et al., 2022; Muto et al., 2024; Polisetty et al., 2024; Rawindaran et al., 2021; Roux et al., 2023; Saleem et al., 2023)

## Discussion

The results of this study provide compelling evidence of AI’s transformative impact on SMEs, with significant implications for innovation, managerial decision-making, and strategic competitiveness. By integrating insights from a B-SLR, the study identifies key thematic areas, prevailing methodological trends, and existing research gaps, offering a comprehensive understanding of AI adoption in SMEs. The bibliometric analysis indicates a marked increase in scholarly attention to AI adoption in SMEs since 2020, reflecting global shifts toward digital transformation, Industry 4.0, and the growing recognition of AI as a strategic enabler ( Khan et al., 2025; Schwaeke et al., 2025). This trend suggests that SMEs are increasingly positioned at the intersection of technological innovation and entrepreneurship, leveraging AI to enhance operational efficiency and create strategic value. The identified thematic clusters, AI-driven innovation, entrepreneurial decision-making, and adoption barriers/enablers, highlight the multidimensional nature of AI integration within SMEs ( Upadhyay et al., 2023; Wang et al., 2022).

AI-driven innovation emerges as a central outcome of adoption. Literature consistently shows that SMEs leveraging AI technologies experience enhanced product and process innovation, faster time-to-market, and improved operational efficiency ( Cooper, 2025). These findings align with the dynamic capabilities perspective, which emphasizes the importance of leveraging technology to sense and seize market opportunities, reconfigure resources, and achieve a sustainable competitive advantage ( Drydakis, 2022). SMEs that integrate AI can anticipate market shifts, customize their offerings, and optimize resource allocation, thereby positioning themselves as agile and innovative actors within competitive environments ( Carayannis et al., 2025). This underscores the strategic potential of AI adoption, enabling SMEs to overcome traditional resource constraints by harnessing intelligent technologies.

The study also highlights the role of AI in supporting entrepreneurial decision-making. Predictive analytics, data-driven insights, and decision-support systems enhance the capacity of SMEs to identify business opportunities, evaluate risks, and make informed strategic choices ( Žilka et al., 2024). This indicates that AI functions not only as a technological tool but also as an enabler of managerial cognition and strategic foresight. By facilitating evidence-based decisions, AI adoption allows SMEs to navigate uncertainty, respond effectively to market disruptions, and align entrepreneurial initiatives with emerging business trends ( Siddiqui et al., 2024). Consequently, AI adoption is intricately linked to the development of entrepreneurial capabilities and fostering resilience and adaptability in small, resource-constrained organizations.

Despite these benefits, adoption is shaped by a complex interplay of internal and external factors. Organizational readiness, encompassing technological infrastructure, managerial expertise, and digital literacy, is crucial in determining the success of adoption ( Adam & Alarifi, 2021; Kumar & Sharma, 2023). SMEs often face barriers such as high implementation costs, a lack of skilled personnel, and limited awareness of AI applications ( Al Dhaheri et al., 2024). Externally, regulatory frameworks, industry standards, and access to collaborative networks influence the extent and impact of AI adoption ( Arroyabe et al., 2024; Chatterjee, 2020). These findings suggest that adoption is not a linear process, but rather requires tailored strategies that account for the unique contexts of SMEs across different regions and sectors.

From a methodological perspective, the study relies strongly on case studies and surveys, which provide rich qualitative and quantitative insights but limit longitudinal understanding of AI adoption outcomes ( Bedué & Fritzsche, 2022; Polisetty et al., 2024; Uren & Edwards, 2023). The scarcity of longitudinal, experimental, and mixed-method studies represents a significant gap, particularly in understanding the sustained impact of AI on innovation performance, financial outcomes, and strategic decision-making. Additionally, the research concentration in developed economies limits generalizability to SMEs in emerging markets, where resource constraints, infrastructure limitations, and contextual challenges may influence adoption trajectories differently ( Ashiru et al., 2023; Elbanna et al., 2020). Therefore, targeted empirical studies in underrepresented regions and diverse sectors are crucial for a comprehensive understanding of AI adoption globally in SMEs.

Synthesizing B-SLR findings highlights strategic research opportunities. Future studies could examine sector-specific AI adoption pathways, particularly in service and non-manufacturing SMEs, where digital transformation is increasingly critical. Longitudinal and experimental designs are necessary to assess the sustained impact of AI on innovation, decision-making, and firm performance. Research in emerging economies can shed light on the role of institutional support, funding mechanisms, and local entrepreneurial ecosystems in facilitating the adoption of innovative solutions. Ultimately, integrating multi-level analyses that consider individual, organizational, and environmental determinants can yield nuanced insights, thereby advancing both theory and practice.

AI adoption in SMEs is a multidimensional phenomenon shaped by technological capabilities, managerial competencies, organizational culture, and environmental factors. Strategic AI integration fosters innovation, supports entrepreneurial decision-making, and enhances competitive resilience, while challenges persist regarding resource, knowledge, and institutional support. These insights offer significant implications for scholars, practitioners, and policymakers aiming to transform SMEs into agile, innovative, and competitive entities in rapidly evolving business environments.

## Future directions and implications

The findings of this study highlight several critical directions for advancing research on AI adoption in SMEs. First, there is a clear need for longitudinal studies to assess the sustained impact of AI on organizational performance, innovation outcomes, and entrepreneurial decision-making. Much existing research relies on cross-sectional designs, which overlook dynamic adoption trajectories and long-term effects. Longitudinal approaches would generate richer insights into how SMEs evolve with AI integration, shedding light on how technological adoption fosters strategic agility, resilience, and sustained competitiveness ( Ayinaddis, 2025).

Second, research should explore sector-specific pathways of AI adoption. While manufacturing SMEs dominate existing studies, service-oriented, retail, and creative industries remain significantly underexplored ( Wang & Zhang, 2025). Examining AI integration across diverse sectors will illuminate how technological adaptation, organizational processes, and innovation outputs differ in various contexts, offering nuanced guidance for managers seeking to leverage AI for value creation. This sectoral lens is critical as AI adoption increasingly intersects with digital services, customer experience, and knowledge-intensive business models.

Third, there is a critical need for contextualized research in emerging economies. Most existing studies focus on SMEs in Europe and North America, leaving a substantial knowledge gap regarding adoption barriers, institutional support, and innovation outcomes in Africa, Latin America, and Asia ( Balzano et al., 2025; Torres de Oliveira et al., 2022). Comparative studies across regions can reveal how infrastructural limitations, regulatory environments, cultural factors, and entrepreneurial ecosystems shape AI adoption and its outcomes. Such research would broaden the generalizability of the findings and provide actionable insights for policymakers and international development agencies seeking to enhance SME competitiveness in resource-constrained environments.

Future research should adopt a multi-level approach to better understand the adoption of AI by SMEs. This involves examining factors at the individual level (such as managerial skills and employee AI literacy), the organizational level (including culture and resources), and the environmental level (including knowledge networks and regulations). Considering these factors together would provide a more comprehensive picture of how AI adoption creates value ( Dwivedi et al., 2021). Using experimental or mixed-method approaches would also improve causal insights, helping researchers link AI adoption more directly to innovation, decision-making, and firm performance.

Theoretically, this study reinforces and extends the dynamic capabilities framework by demonstrating that AI serves as a strategic resource, enabling SMEs to sense opportunities, seize market advantages, and reconfigure internal capabilities for innovation and competitiveness ( Teece, 2018). The findings highlight the mediating role of organizational readiness, technological infrastructure, and external support mechanisms. This demonstrates that successful adoption is not purely a function of technology but emerges from the interplay of human, organizational, and environmental factors. These insights advance technology adoption, innovation management, and entrepreneurship theory by highlighting the complex interdependencies that shape the adoption of AI in SMEs.

Practically, this study provides guidance to managers on how to enhance the effectiveness of AI adoption. Aligning AI with organizational goals is crucial to unlock its full potential for innovation, improved decision-making, and a competitive advantage, beyond simply enhancing efficiency. Building capacity through employee training, enhancing managerial AI literacy, and hiring skilled personnel are key steps for successful use. SMEs should also evaluate their technological infrastructure, digital maturity, and internal processes to ensure readiness. Additionally, collaborating with industry partners and leveraging external knowledge networks can help overcome adoption barriers and enhance innovation outcomes.

Policymakers also play a pivotal role in enabling AI adoption. Tailored policies addressing SMEs’ financial constraints, limited technical expertise, and digital infrastructure gaps can facilitate adoption and maximize their benefits. Initiatives such as tax incentives, subsidized training programs, and regional innovation hubs can provide both the resources and guidance SMEs need. In parallel, clear regulatory frameworks for data privacy, AI ethics, and compliance standards can help reduce uncertainty, fostering the responsible and sustainable integration of AI. By addressing both organizational and institutional barriers, such policies can accelerate SMEs’ transformation into agile, innovative, and competitive enterprises.

In summary, future research should use longitudinal, sector-specific, and context-sensitive approaches, combining multi-level and experimental designs to capture both the complexity and long-term effects of AI adoption in SMEs. Theoretically, studies should focus on how technology, managerial capabilities, and environmental factors interact with each other. Practically, attention should be given to strategic integration, capacity building, and supportive ecosystems. These directions can help SMEs, scholars, and policymakers harness AI for sustainable innovation, agility, and competitiveness in a digital economy. Policymakers and government agencies also play a crucial role in promoting the adoption of AI. Tailored policies are necessary to address the challenges faced by SMEs, including financial constraints, limited expertise, and inadequate digital infrastructure. Possible measures include tax incentives for AI investment, subsidized training, and the establishment of regional innovation hubs. Clear regulations on data privacy, AI ethics, and compliance can further reduce uncertainty and support the responsible adoption of AI.

## Conclusion

This study provides a comprehensive and evidence-based understanding of AI adoption in SMEs by integrating bibliometric analysis with a SLR. The bibliometric analysis quantitatively maps the growth of research, influential institutions, thematic clusters, and intellectual networks, revealing a rapidly expanding field concentrated primarily in developed economies. Complementing this, the SLR synthesizes empirical evidence on AI-driven innovation, entrepreneurial decision-making, and adoption barriers and enablers, collectively offering a holistic view of the current state of knowledge while identifying critical gaps that warrant scholarly attention.

The findings highlight that AI adoption serves as a strategic enabler for SMEs, strengthening innovation capabilities, supporting evidence-based decision-making, and enhancing operational efficiency. When effectively leveraged, AI enables SMEs to overcome resource constraints, respond proactively to changing market conditions, and establish a sustainable competitive advantage. However, successful adoption requires organizational readiness, managerial expertise, a robust technological infrastructure, and a supportive external environment. Methodologically, the dominance of case studies and surveys underscores the need for more longitudinal, experimental, and mixed-method research to capture the long-term and context-specific impacts of AI adoption.

Geographical disparities in the literature point to an urgent need for research in emerging economies, where SMEs face distinct challenges and opportunities. Policymakers can accelerate adoption by providing targeted support programs, investing in infrastructure development, and offering digital skills training. At the same time, SME managers can enhance organizational readiness, workforce capabilities, and strategic integration of AI into decision-making processes. For scholars, this study identifies important future research directions, including the exploration of sector-specific adoption patterns, examination of long-term performance outcomes, and investigation of how technological, organizational, and environmental factors interact.

In conclusion, this study makes significant contributions to both theory and practice by offering a holistic understanding of AI adoption in SMEs. It clarifies the intellectual structure, thematic priorities, methodological trends, and research gaps in the field while providing actionable insights for managers and policymakers. By combining bibliometric and systematic review approaches, this study not only maps the current body of knowledge but also provides a foundation for future research and evidence-based interventions to promote innovation, competitiveness, and sustainable growth in SMEs worldwide.

## Data Availability

The datasets used in this study are publicly accessible through the Zenodo repository under the title Data Repository Scopus 63, with the working DOI https://doi.org/10.5281/zenodo.17375011 ( Yesuf, 2025). Data are available under the terms of the Creative Commons Attribution 4.0 International license (CC-BY 4.0).
