# Supplementary material for: Artificial Intelligence Adoption as a Driver of Innovation and Competitiveness in SMEs: A Bibliometric and Systematic Review
Source: F1000Res. 2025 Oct 31;14:1187. [Version 1] doi: 10.12688/f1000research.171494.1 (PMC12749562; doi:10.12688/f1000research.171494.1)
Supplement: Peer review history

# Reviewer report: Reviewer response for version 1

**DOI:** 10.5256/f1000research.189110.r431707

**Published:** 2025-12-29

**Authors:** Arjita Jain

**Affiliations:** 1 NCRD's Sterling Institute of Management Studies,, Navi Mumbai, India

This study provides strong evidence of AI’s growing and transformative role in SMEs, particularly in innovation, decision-making, and competitive strategy. Drawing on a B-SLR, the authors map key themes, methodological patterns, and research gaps, offering a clear overview of current scholarship on AI adoption in SMEs.

 The bibliometric analysis shows a sharp rise in academic interest after 2020, mirroring global moves toward digital transformation and Industry 4.0. This surge underscores AI’s emergence as a strategic tool for SMEs seeking operational efficiency and value creation.

 Overall, the study’s identification of major thematic clusters—AI-driven innovation, entrepreneurial decision-making, and adoption barriers and enablers—highlights the complex and multidimensional nature of AI integration within the SME sector.

Are the rationale for, and objectives of, the Systematic Review clearly stated?

Yes

Is the statistical analysis and its interpretation appropriate?

Yes

If this is a Living Systematic Review, is the ‘living’ method appropriate and is the search schedule clearly defined and justified? (‘Living Systematic Review’ or a variation of this term should be included in the title.)

Yes

Are sufficient details of the methods and analysis provided to allow replication by others?

Yes

Are the conclusions drawn adequately supported by the results presented in the review?

Yes

Reviewer Expertise:

Reimagining Management Education, Education  4.o, SMEs, CBHRM, Employee Engagement, NEP 2020 etc

I confirm that I have read this submission and believe that I have an appropriate level of expertise to confirm that it is of an acceptable scientific standard.

# Reviewer report: Reviewer response for version 1

**DOI:** 10.5256/f1000research.189110.r429211

**Published:** 2025-11-08

**Authors:** Erstu kassa

**Affiliations:** 1 Woldia University, Weldiya, Amhara, Ethiopia

The review topic is interesting and contemporary.

 My comments and suggestions are;

 1. Please remove the citation in table 3. 

 2. In the abstract section, remove numbers (1), (2), and (3).

 3. The introduction section is well written.

 4. Add a separate topic for the literature review. 

 5. The method section is good.

Are the rationale for, and objectives of, the Systematic Review clearly stated?

Yes

Is the statistical analysis and its interpretation appropriate?

Not applicable

If this is a Living Systematic Review, is the ‘living’ method appropriate and is the search schedule clearly defined and justified? (‘Living Systematic Review’ or a variation of this term should be included in the title.)

Yes

Are sufficient details of the methods and analysis provided to allow replication by others?

Yes

Are the conclusions drawn adequately supported by the results presented in the review?

Yes

Reviewer Expertise:

Knowledge management, digital transformation, strategic management

I confirm that I have read this submission and believe that I have an appropriate level of expertise to confirm that it is of an acceptable scientific standard.
